# The Histone Deacetylases MoRpd3 and MoHst4 Regulate Growth, Conidiation, and Pathogenicity in the Rice Blast Fungus Magnaporthe oryzae

**DOI:** 10.1128/mSphere.00118-21

**Published:** 2021-06-30

**Authors:** Chaoxiang Lin, Xue Cao, Ziwei Qu, Shulin Zhang, Naweed I. Naqvi, Yi Zhen Deng

**Affiliations:** a State Key Laboratory for Conservation and Utilization of Subtropical Agro-Bioresources, Guangdong Province Key Laboratory of Microbial Signals and Disease Control, Integrative Microbiology Research Centre, South China Agricultural University, Guangzhou, China; b Laboratory of Plant Virology, Department of Plant Pathology, College of Plant Protection, South China Agricultural University, Guangzhou, China; c Temasek Life Sciences Laboratory, National University of Singapore, Singapore; d Department of Biological Sciences, National University of Singapore, Singapore; e Department of Plant Pathology, College of Plant Protection, Anhui Agricultural University, Hefei, China; Carnegie Mellon University

**Keywords:** Hst4, *Magnaporthe oryzae*, pathogenesis, Rpd3, Sin3, HDAC, histone deacetylation complex

## Abstract

As the causal agent of the blast disease, Magnaporthe oryzae is one of the most destructive fungal pathogens of rice. Histone acetylation/deacetylation is important for remodeling of chromatin superstructure and thus altering gene expression. In this study, two genes encoding histone deacetylases, namely, *MoRPD3* and *MoHST4*, were identified and functionally characterized in M. oryzae. MoHst4 was required for proper mycelial growth and pathogenicity, whereas overproduction of MoRpd3 led to loss of pathogenicity, likely due to a block in conidial cell death and restricted invasive growth within the host plants. Green fluorescent protein (GFP)-MoRpd3 localized to the nucleus and cytoplasm in vegetative hyphae and developing conidia. By comparative transcriptomics analysis, we identified potential target genes epigenetically regulated by histone deacetylases (HDACs) containing MoRpd3 or MoHst4, which may contribute to conidia formation and/or conidial cell death, which is a prerequisite for successful appressorium-mediated host invasion. Taken together, our results suggest that histone deacetylases MoRpd3 and MoHst4 differentially regulate mycelial growth, asexual development, and pathogenesis in M. oryzae.

**IMPORTANCE** HDACs (histone deacetylases) regulate various aspects of growth, development, and pathogenesis in plant-pathogenic fungi. Most members of HDAC classes I to III have been functionally characterized, except for orthologous Rpd3 and Hst4, in the rice blast fungus Magnaporthe oryzae. In this study, we assessed the function of MoRpd3 and MoHst4 by reverse genetics and found that they differentially regulate *M. oryzae* vegetative growth, asexual development, and infection. Particularly, MoRpd3 negatively regulates *M. oryzae* pathogenicity, likely through suppression of conidial cell death, which we recently reported as being critical for appressorium maturation and functioning. Overall, this study broadens our understanding of fungal pathobiology and its critical regulation by histone modification(s) during cell death and *in planta* differentiation.

## INTRODUCTION

Histone acetylation and deacetylation are some of the most well-studied epigenetic modifications, catalyzed, respectively, by histone acetyltransferases (HATs) or histone deacetylases (HDACs) (1). Acetylation is the most prominent modification on core histones that strongly affects nuclear processes such as DNA replication, DNA repair, and DNA transcription (2). It has been reported that epigenetic regulation mediated by histone acetylation/deacetylation plays an important role in pathogenic development in fungi (3–7).

BLAST, caused by the filamentous ascomycete Magnaporthe oryzae, is a serious disease of rice and other cereal crops. M. oryzae produces asexual spores or conidia, which sense suitable signals/cues from the host surface to differentiate into dome-shaped structures named appressoria, which assist in host penetration and infection (8–10). Protein acetylation catalyzed by histone acetylases or deacetylases plays important regulatory roles in this fungal infection process. The catalytic subunits of HDACs identified in *M. oryzae* are divided into three major classes: class I, Rpd3 and Hos2, class II, Hda1 and Hos3, and class III, Sirt1, Sir2 (silent information regulator 2), Hst1, Sirt3/Hst4, Sirt4, and Sirt5 (11, 12). A number of HDACs have been functionally characterized in *M. oryzae* (13–18). Treatment with an HDAC inhibitor targeting Rpd3/Hda1 family suppresses appressorium differentiation in *M. oryzae* (13). *MoHDA1* and *MoHOS2* are required for vegetative growth and conidiation, and *MoHOS2* is required for appressorium formation (13, 14). Deletion of the gene encoding an Hda1-type histone deacetylase led to elevated accumulation of the shunt products of the 1,8-dihydroxynaphthalene and the ergosterol pathways and the transcriptional activation of the three melanin biosynthesis genes (15). The histone deacetylase MoSir2 inhibits the host immune response by regulating the expression of a superoxide dismutase (SOD) gene and is critical for the invasive *in planta* growth of *M. oryzae* (16). The TIG1 HDAC was shown to be necessary for vegetative growth, conidia production, and pathogenicity of *M. oryzae* (17). *MoSNT2* was found to regulate autophagy in *M. oryzae* by regulating the acetylation state of histone H3, thus affecting infection (18).

*RPD3* encodes a catalytic component of the class I HDACs. Yeast Rpd3 plays a role in removing acetylation from core histones H3 and H4 and regulates gene expression and heterochromatin silencing (19). Yeast *HST4* belongs to Sir2 family of class III HDACs and contributes to proper cell cycle progression, radiation resistance, and genomic stability, likely via regulation of telomeric silencing (20). In addition to deacetylating histone proteins, Hst4/Sirt3 deacetylase was shown to catalyze the deacetylation of mitochondrial substrates in yeast (21) or human cell lines (22) and to regulate cellular respiration and redox levels (21, 23, 24). In mammals, Sirt3 functions as a tumor suppressor, and mice lacking Sirt3 are more prone to cancer development (25). However, the orthologous Rpd3 and Hst4 in *M. oryza*e have not been characterized.

In this study, we identified two genes, *MoRPD3* and *MoHst4*, encoding histone deacetylases in *M. oryzae*. Deletion of *MoHst4* led to a significant reduction in mycelial growth and conidiation. Deletion of *MoRPD3* was unsuccessful despite repeated attempts, suggesting that this gene may be essential for viability. However, loss of Rpd3-associated Sin3 function caused severely restricted mycelial growth and asexual development. In contrast, overexpression of *MoRPD3* caused increased conidia formation, decreased production of infection hyphae, and loss of pathogenicity. GFP-MoRpd3 was found to localize to the nucleus and cytoplasm in vegetative hyphae and conidia. Our data demonstrate that *MoRPD3* and *MoHST4* are required for proper conidiogenesis and infectious growth in rice blast.

## RESULTS

### Generation of *MoHST4* and *MoRPD3* mutants in *M. oryzae*.

The *M. oryzae* proteins MoHst4 (EHA53805) and MoRpd3 (EHA51874) were previously identified and classified as class III and class I histone deacetylases (12), yet their biological functions have not been characterized. We monitored their expression pattern during asexual and pathogenic development by real-time quantitative PCR (qRT-PCR). The results showed that the transcriptional level of *MoHST4* was significantly (*P* < 0.05) reduced in developing conidia and appressoria in comparison to that in the vegetative mycelia (Fig. S1A). In contrast, *MoHST4* transcript was significantly (*P* < 0.05) upregulated at 12 h postinfection in the rice plants (Fig. S1A). On the other hand, *MoRPD3* appeared stably transcribed in mycelia, conidia, and appressoria, while it was significantly induced in early stages of invasive growth (12 hours post infection [hpi]) (Fig. S1A). Transcription of both *MoHST4* and *MoRPD3* returned to a level comparable to that in the mycelia at 24 h postinfection (Fig. S1A).

10.1128/mSphere.00118-21.1 FIG S1 Generation and verification of the strains used in this study. (A) Gene expression patterns of *MoHST4* and *MoRPD3* were assessed by qRT-PCR during vegetative growth, conidiation appressorium formation, and invasive growth. Relative fold change was calculated by the 2^−ΔΔCt^ method. *ACTIN* gene was used as an internal control. Means and standard deviations were calculated based on three independent experiments, each of which contained three technical replicates. *, *P* < 0.05 versus mycelia. (B) Schematic diagram of the genomic region of the *MoHST4* in *M. oryzae* and replacement by *HPH* by homologous recombination occurring on the flanking regions. Relevant restriction enzyme sites distributed on the *MoHST4* locus or on the deletion constructs are denoted. The PstI-HindIII fragment (5′-UTR) was used as the probe for Southern blotting as shown in panel C. (C) Southern blotting to verify the knockout and complementation of *MoHST4* gene. Genomic DNA of indicated strains were digested by *Hind*III and *Kpn*I. The presence of the 1.4-kb band in the WT and its absence in the deletion mutant, together with the occurrence of the 2.8-kb band (arrow), confirmed the successful replacement of *MoHST4* locus with *HPH^+^.* The same 2.8-kb band could be detected in the genetic complementation strain *MoHST4c*, as it was generated in the *mohst4*Δ mutant background. An extra band (*) detected by the probe (corresponding to the promoter region of *MoHST4*) indicated insertion of the *MoHST4*-eGFP fusion construct into the *mohst4*Δ genome. (D) qRT-PCR detected the transcription level of *MoRPD3* in the wild-type and *RPD3* overexpression strain. The endogenous *MoRPD3* gene transcription level was set as “1.” Relative fold change was calculated using the 2^−ΔΔCt^ method, with *ACTIN* gene as an internal control. Mean ± SE was derived from three independent experiments, each containing three technical replicates. ***, *P* < 0.001 versus WT. (E) Total protein lysates from WT or *RPD3OX* strains were analyzed by immunoblotting with anti-GFP antibodies (mouse; 1:5,000; Proteintech, 66002-1-lg). Arrow denotes the GFP-Rpd3 fusion proteins. Molecular weight (kDa) as indicated. (F) Immunoblotting assay using total proteins isolated from the wild type (WT), *MoRPD3OX*, or *mohst4*Δ mutant, using the anti-lysine acetylated (AcK) antibody (rabbit; 1:1,000; Abcam, ab61257) or anti-acetyl-histone H3 (H3AcK) antibody (rabbit; 1:1,000; Active Motif, 61937). Coomassie blue staining of the total protein served as loading control. Download FIG S1, TIF file, 1.1 MB. Copyright © 2021 Lin et al. 2021 Lin et al. https://creativecommons.org/licenses/by/4.0/ This content is distributed under the terms of the Creative Commons Attribution 4.0 International license.

Next, we attempted to generate targeted gene deletion mutants for *MoHST4* (*MGG_04588*) and *MoRpd3* (*MGG_05857*) by homologous recombination. We obtained two *mohst4*Δ mutants, as verified by Southern blotting (Fig. S1B and C). The complementation strain *MoHST4c* was also generated, by introducing the full-length *MoHST4* genomic locus into the *mohst4*Δ mutant (Fig. S1C, lane 1). However, our repeated attempts to generate a *MoRPD3* deletion mutant failed, thus suggesting its importance for viability of *M. oryzae*. Instead, we generated two overexpression strains of *MoRPD3*, named *RPD3OX* (OX), in order to investigate its cellular function. A GFP-MoRpd3 fusion protein was expressed under the constitutive *RP27* promoter in the *RPD3OX* strain (details in Materials and Methods). Two *RPD3OX* mutants were verified by qRT-PCR, respectively displaying approximately 15- and 12-fold upregulation of the *RPD3* transcript levels compared to those of the wild type (Fig. S1D). Expression of GFP-MoRpd3 fusion protein was verified by immunoblotting (Fig. S1E).

Furthermore, immunoblotting with an anti-AcK antibody (Abcam, ab61257) confirmed that the acetylation levels of the target proteins were markedly reduced in the *RPD3OX* strain and increased in the *mohst4*Δ mutant (Fig. S1F). We further tested the levels of H3 acetylation by using an anti-H3AcK antibody (Active Motif, 61937) in the aforementioned three strains. Similarly, H3 acetylation was reduced in the *RPD3OX* strain and noticeably higher in the *mohst4*Δ mutant (Fig. S1F). We conclude that both MoHst4 and MoRpd3 are functional protein deacetylases, able to remove acetylation from proteins, including the histone H3.

### *MoHST4* and *MoRPD3* regulate *M. oryzae* mycelial growth and conidiation.

**FIG 1:**
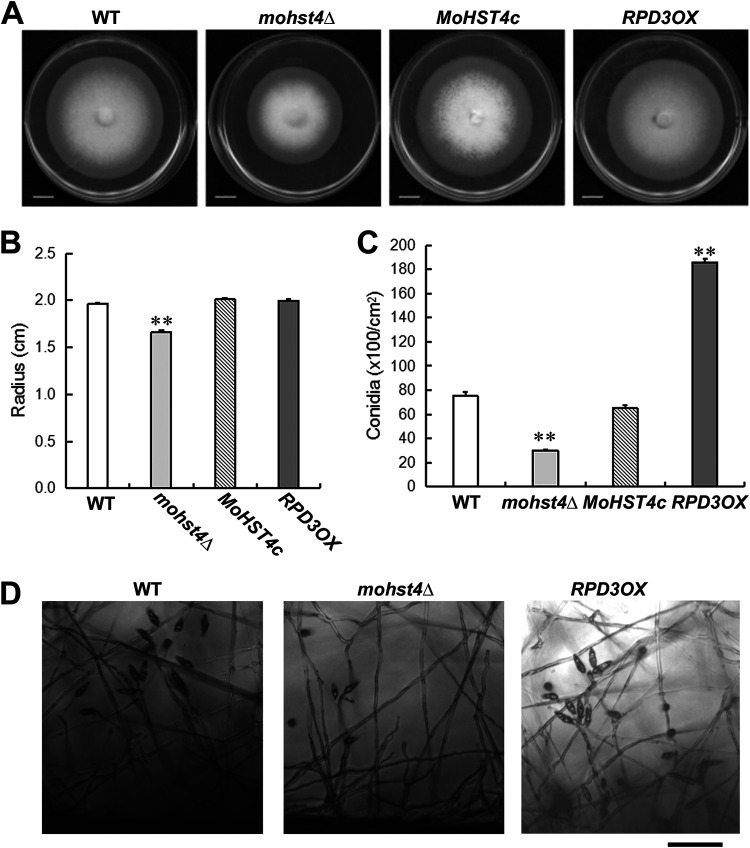
Vegetative growth and asexual development of the *mohst4*Δ and the *RPD3OX* strain. (A) WT, the *mohst4*Δ mutant, complementation strain (*MoHST4c*), and the *RPD3OX* mutant grew on PA medium at 28°C for 7 days before they were photographed. Scale bar represents 1 cm. (B) Colony radii were measured at 7 dpi. Mean ± standard error (SE) values were derived from four independent biological repeats. **, *P < *0.01 versus WT. (C) Bar chart presentation of conidia produced by each strain. Mean ± SE values were derived from four independent biological repeats. **, *P < *0.01 versus WT. (D) Photomicrographs depicting conidia formation in aforementioned strains. Images were taken 48 h post induction of conidiation. Scale bar represents 10 μm.

To characterize the biological functions of *MoHST4* and *MoRPD3*, we first assessed vegetative growth of *mohst4*Δ mutant and *MoRPD3OX*. Colony morphology and radial growth were assessed in these strains cultured on prune agar (PA) medium at 28°C for 7 days. The results showed that, compared to that of the wild-type (WT) strain, the *mohst4*Δ mutant displayed significantly (*P* < 0.01) reduced radial growth (Fig. 1A and B). Genetic complementation of *MoHST4* gene (*MoHST4c* strain) was able to fully suppress the phenotypic defects of the *mohst4*Δ (Fig. 1A and B). On the other hand, no obvious vegetative growth defects were observed in the *MoRPD3OX* strain (Fig. 1A and B). We conclude that *MoHST4* is important for mycelial growth, while overexpression of *MoRPD3* gene does not affect vegetative development in *M. oryzae*.

Next, we examined the production of asexual spores, conidia, of the respective WT, mutant, and OX strains. We found that conidiation in the *mohst4*Δ mutant was significantly (*P* < 0.01) reduced in comparison to that in the WT or the complemented *MoHST4c* strain (Fig. 1C). We noticed that in comparison to those in the WT, fewer conidiophores bearing conidia were evident in the *mohst4*Δ mutant (Fig. 1D). In contrast, the *RPD3OX* strain produced many more conidia than did the WT (*P* < 0.01) (Fig. 1C), and we observed that more conidia were formed on a single conidiophore in this strain background (Fig. 1D). Overall, our results indicate that both MoHst4 and MoRpd3 positively regulate *M. oryzae* conidiation.

### *MoHST4* and *MoRPD3* function in *M. oryzae* pathogenicity.

**FIG 2:**
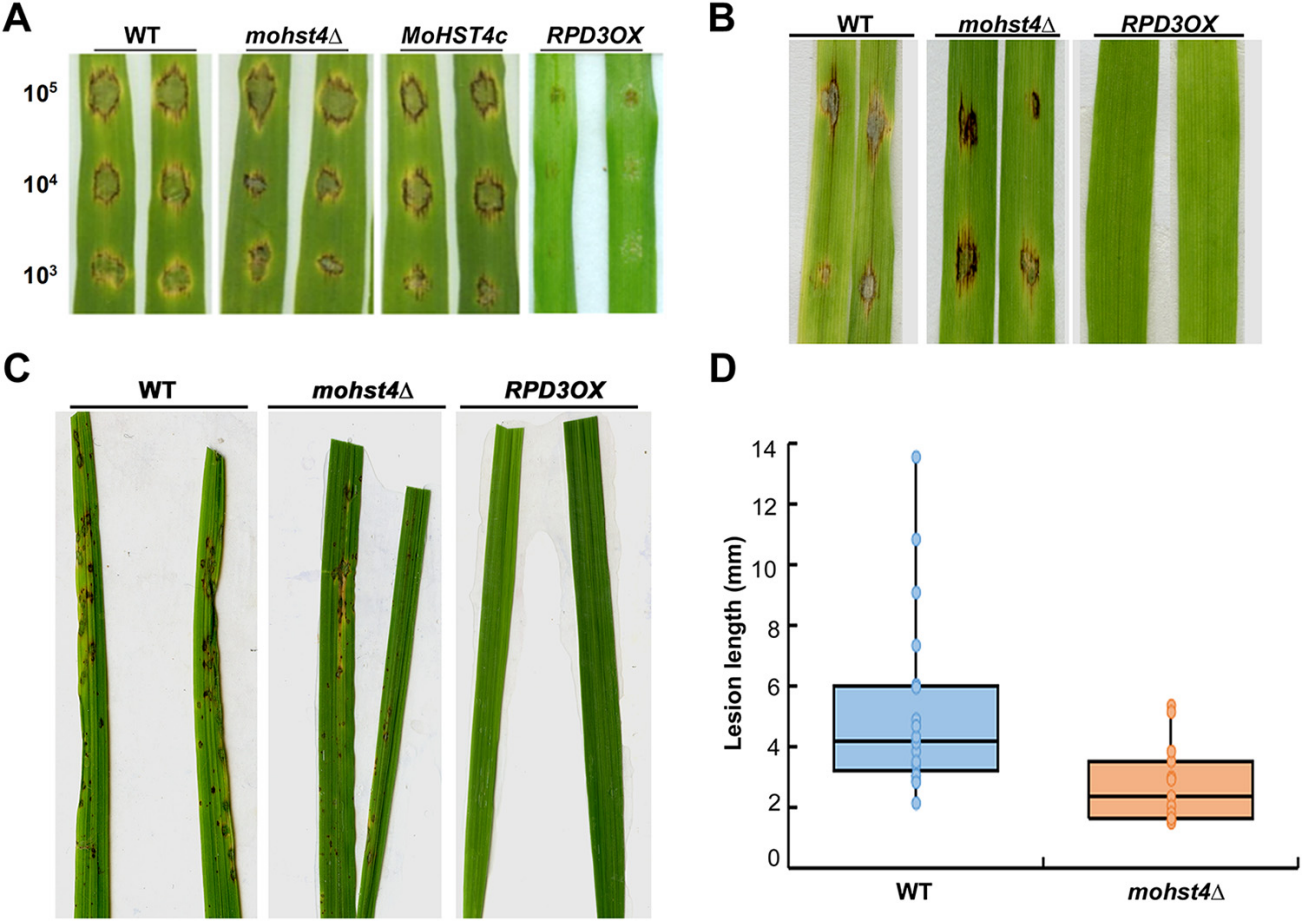
*MoRPD3* negatively regulates *M. oryzae* pathogenicity. Pathogenicity at different conidia concentrations was evaluated on detached barley leaves, intact (A) or with wounding (B). The inoculated leaves were kept in a high humidity (>90%) at 25°C in darkness for the first 24 h, followed by a 16 h:8 h light:dark cycle. Photographs were taken at 7 dpi. (C) Infection assay using rice seedlings. Conidial suspension (2 × 10^5^ conidia/ml) of the indicated strains were sprayed to the rice seedlings (5 ml conidial suspension for each pot containing 6 seedlings). The rice leaves were cut and photographed at 7 dpi. The experiments were repeated three times and representative images were displayed. (D) Quantification of lesion number and size based on the rice seedling infection assays presented in panel B. Mean ± SE values were derived from 9 leaves across three biological repeats in each instance.

To evaluate the role of *MoHST4* and *MoRPD3* in pathogenicity, we first carried out infection assays by droplet inoculation of conidial suspensions on detached barley leaves. The WT, the *mohst4*Δ mutant, and the mutant’s complemented strain showed typical blast lesions after 5 days postinoculation (dpi), while the *RPD3OX* strain was nonpathogenic (Fig. 2A). We further tested whether the *RPD3OX* conidia were able to infect the detached barley leaves through wounds. The result showed that on the wounded leaves, there were still no disease lesions caused by the *RPD3OX* conidia (Fig. 2B).

We also performed blast infection assay by spraying the conidia suspension (2 × 10^5^ to 3 × 10^5^ cell/ml) onto the rice seedlings. The results showed that numerous blast lesions were formed on the rice leaves sprayed with WT conidia (5 to 7 dpi) (Fig. 2C). According to the “standard evaluation system for rice leaf blast” (26), predominant lesions caused by the WT conidia were of type code 5 to 7, as their length was more than 3 mm and sometimes coalescing, infecting an estimated 4.78 to 16.48% of the total leaf area. The conidia of *RPD3OX* strain caused no lesions at all on the rice leaves (Fig. 2C). The *mohst4*Δ could cause leaf blast lesions on the rice seedlings (Fig. 2C), but the predominant lesion length was less than 3 mm, infecting less than 4% of the leaf area, and therefore of the type code 1 to 3 (Fig. 2D). Overall, we conclude that MoRpd3 negatively regulates *M. oryzae* pathogenicity and MoHst4 is required for full pathogenicity on rice seedlings.

**FIG 3:**
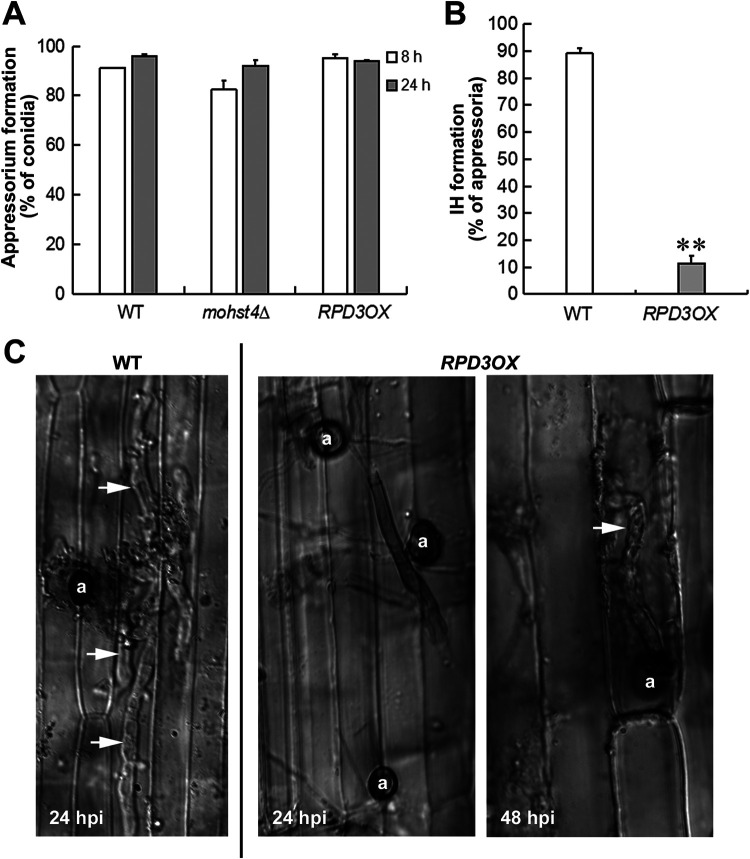
*MoRPD3* negatively regulates invasive growth during infection. (A) Appressorium formation rates of WT, the *mohst4*Δ mutant, and the *RPD3OX* mutant were quantified at 8 hpi or 24 hpi on inductive surface. Mean ± SE values were derived from four independent biological repeats, each of which contained >300 conidia for each instance. (B) Invasive hyphal growth was quantified at 24 hpi on rice leaf sheath. Data are presented as mean ± SE derived from three independent biological repeats containing >50 conidia in each instance. ** denotes significant difference (*P < *0.01). (C) Invasive hyphae were observed and photographed at 24 or 48 hpi. Scale bar represents 10 μm. Arrows denote invasive hypha. a, appressorium.

We were particularly interested to find out the reason for loss of pathogenicity upon overproduction of the MoRpd3 and therefore initially assessed the ability of the *RPD3OX* conidia to germinate and develop appressoria on the artificial inductive surface. Our results showed that the appressorium formation rate was comparable in the *RPD3OX*, *mohst4*Δ, and WT strains at 8 or 24 hpi (Fig. 3A). Therefore, we infer that the *RPD3OX* conidia are not defective in appressorium formation but likely are in other step(s) of infection.

We further performed appressorium-based host penetration assays using rice leaf sheath to examine the invasive growth of the WT and *RPD3OX* strain. We observed that a high percentage (close to 90%) of WT appressoria could form penetration/invasive hyphae (IH) at 24 hpi (Fig. 3B), some of which had already spread to the neighboring cells in the host plant (Fig. 3C). Under the same condition, less than 20% of the *RPD3OX* appressoria developed IH *in planta* at 24 hpi (Fig. 3B). Even extended incubation to 48 hpi did not enable the formation of IH from the *RPD3OX* appressoria, and they were all restricted in the first infected plant cell, thus rendering it unable to spread to the neighboring cells within the sheath (Fig. 3C). Overall, we conclude that *MoRPD3* negatively regulates *M. oryzae* pathogenicity, likely due to defective penetration and invasive growth and spread within the host plants.

### Subcellular localization of MoRpd3 and its effect on conidial death.

**FIG 4:**
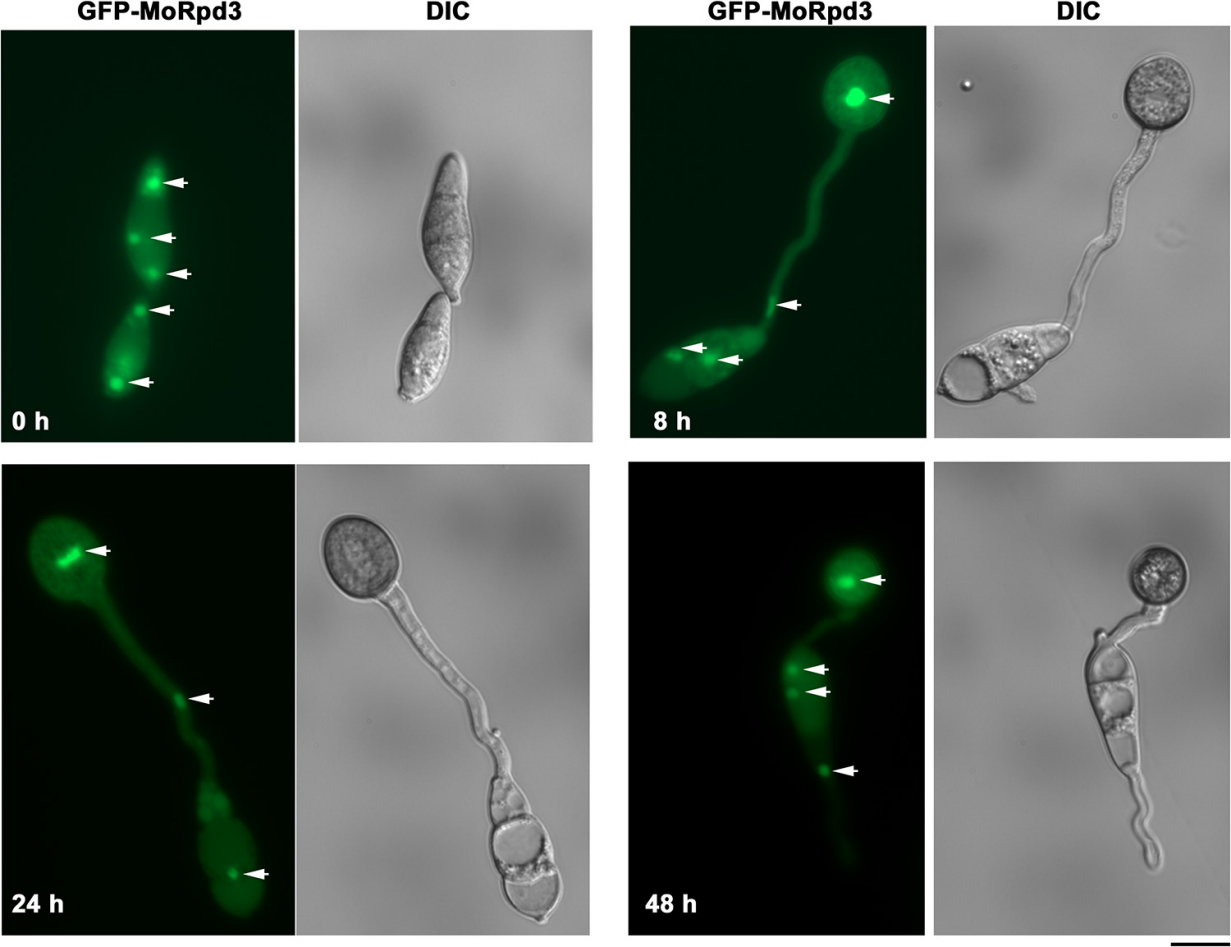
Subcellular localization of GFP-MoRpd3 in *M. oryzae*. Visualization of GFP-MoRpd3 in developing conidia and appressoria at the indicated time points. Scale bar represents 10 μm. Arrows denote nuclei.

**FIG 5:**
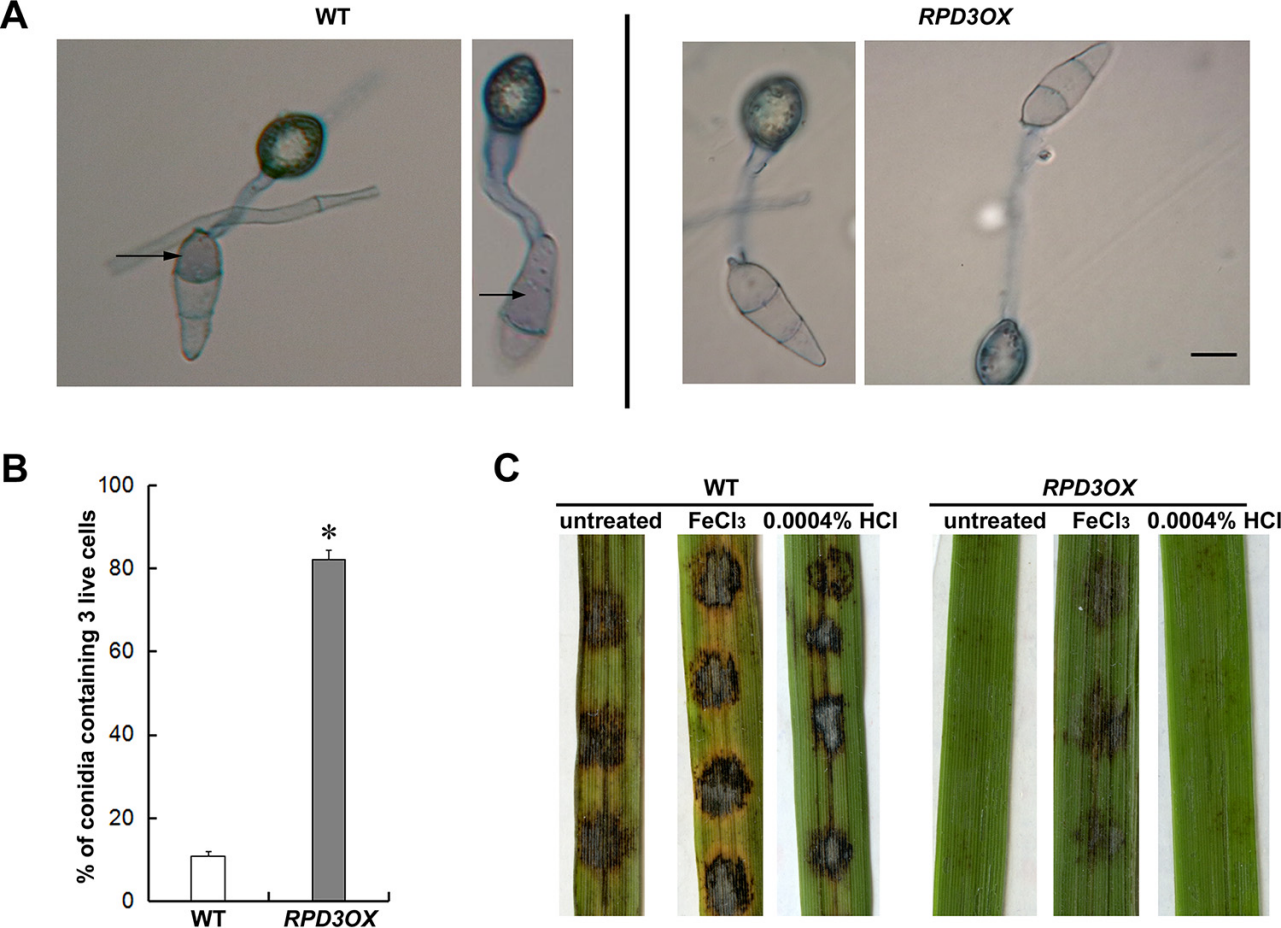
Quantification of conidia viability during appressorium formation/maturation. (A) The WT or the *RPD3OX* conidia were inoculated on the inductive surface for 24 h before trypan blue staining. Arrows denote dead conidia stained by the dye. (B) Quantification of viable conidia at 24 hpi, based on trypan blue staining. Mean ± SE values were derived from three independent biological repeats of the experiment, each of which contained >50 conidia. *, *P < *0.05 versus WT. (C) Conidia of the wild type (WT) or MoRPD3 overexpressed strain (RPD3OX) were inoculated on the detached rice leaves, with or without addition of FeCl_3_ solution (5 mM). 0.0004% HCl served as solvent control. The inoculated leaves were kept in high humidity (>90%) at 25°C in darkness for the first 24 h, followed by a 16 h:8 h light:dark cycle. Photographs were taken at 7 dpi.

As *MoRPD3* negatively regulates *M. oryzae* infection, we explored its subcellular localization by microscopic observation of the GFP-MoRpd3 fusion protein expressed in the *RPD3OX* mutant (Fig. S1E). Under epifluorescence microscope, GFP-MoRpd3 signal was cytosolic and nuclear in conidia at different stages of pathogenic development (Fig. 4). Interestingly, we found that GFP-MoRpd3 persisted in the nuclei and cytosol of the conidia even at a very late stage of appressorium formation (24 or 48 hpi) (Fig. 4), indicating that conidia were still alive or viable. It has been reported that during appressorium formation and maturation, conidial death occurs and is essential for appressorium-mediated host invasion (27, 28). These results led us to infer that conidial death may be blocked/delayed in the *RPD3OX* strain, likely accounting for defective invasive growth in this mutant. To test this hypothesis, we quantified conidial death of the WT and the *RPD3OX* strain by trypan blue staining, following our established method (28). The majority of the WT conidia contained at least one dead cell at 24 hpi (Fig. 5A and B). In contrast, only approximately 20% of the *RPD3OX* conidia experienced such conidial cell death (Fig. 5A and B), thus confirming that MoRpd3 negatively regulates conidial death and consequently suppresses appressorium function and host infection.

We further tested whether addition of FeCl_3_ solution (5 μM), an established inducer of conidial ferroptosis (28), could restore the pathogenicity of *RPD3OX* conidia inoculated on the detached rice leaf. The result showed that exogenous FeCl_3_ promotes lesion formation on the rice leaf by *RPD3OX* strain as well as the WT *M. oryzae* (Fig. 5C). Based on these findings, we conclude that the defective pathogenicity upon overexpression of *MoRPD3* gene was at least partially due to ineffective/lowered conidial ferroptosis.

### Comparative transcriptome analysis of MoHst4 and MoRpd3 mutants.

MoHst4 and MoRpd3 are both catalytic enzymes of HDACs, removing acetylation from histone proteins and thus altering gene expression. With the aim to screen for potential downstream genes that are subject to epigenetic regulation of MoHst4 and/or MoRpd3 during *M. oryzae* conidiation, we performed comparative transcriptome analysis by using total RNA extracted from mycelial cultures under conidiation conditions (details in Materials and Methods). We identified 623 genes in the *mohst4*Δ mutant that were differentially expressed in comparison to those in the WT strain (false-discovery rate [FDR] < 0.05 and |log_2_FC| > 1; three biological repeats), of which 333 genes were upregulated and 290 were downregulated (Data set S1). On the other hand, we identified 858 differentially expressed genes (DEGs) in *RPD3OX* strain versus WT strain, 266 of which were upregulated and 592 of which were downregulated (Data set S1). We noticed that the expression level of the *MoHST4* gene was indeed significantly reduced in the *mohst4*Δ mutant (Data set S1, ID “ncbi_2678115” in sheet named “WTvsKO.significantly different”), while the expression level of *MoRPD3* was significantly upregulated in the *RPD3OX* strain (Data set S1, ID “ncbi_2684118” in sheet named “WTvsOX.significantly different”), both in comparison to the expression levels in the WT strain. This verified that our high-throughput RNA sequencing (RNA-Seq) data were reliable for further gene expression analysis.

10.1128/mSphere.00118-21.6 DATA SET S1 List of differentially expressed genes (DEGs). (Sheet 1) List of DEGs (*P* ≤ 0.05) in the *mohst4*Δ (KO) mutant compared to the wild-type (WT) strain. (Sheet 2) List of DEGs (*P* ≤ 0.05) in the *RPD3OX* (OX) mutant compared to the wild-type (WT) strain. (Sheet 3) The overlapping DEGs of WT versus *mohst4*Δ and WT versus *RPD3OX* (OX). (Sheet 4) The overlapped DEGs from sheet 3, upregulated in the OX strain but downregulated in the KO mutant, and vice versa. Download Data Set S1, XLSX file, 0.3 MB. Copyright © 2021 Lin et al. 2021 Lin et al. https://creativecommons.org/licenses/by/4.0/ This content is distributed under the terms of the Creative Commons Attribution 4.0 International license.

We compared the DEGs in *mohst4*Δ versus WT and *RPD3OX* versus WT, trying to identify common target genes under regulation of both MoHst4 and MoRpd3. Seven genes fall into this category (Data set S1), six of which encode annotated proteins, including 2 hypothetical proteins (MGG_05967, MGG_06799), a chitin binding protein 1 (MGG_05351), a tetratricopeptide repeat domain-containing protein (MGG_08452), a UPF0643 protein (MGG_05157), and a C2H2 zinc-finger transcription factor (MGG_07013). One transcript encoding a TLH5 (telomere-linked helicase) (29) protein was also identified as a common target of MoHst4 and MoRpd3 (Data set S1). These genes may be under regulation by both HDAC I and HDAC III and account for common phenotypes displayed by the *mohst4*Δ and *RPD3OX* mutants.

The DEGs between *mohst4*Δ mutant and the WT strain were highly enriched in membrane components, transporter activity, and ion binding (Fig. S2; Data set S2). On the other hand, the Gene Ontology (GO) enrichment of DEGs between *RPD3OX* and WT strains was in metabolic and developmental process, cellular biosynthetic process, and cytoplasmic components (Fig. S2; Data set S2). KEGG enrichment of DEGs between *mohst4*Δ mutant and the WT strain showed that MoHst4 may regulate multiple metabolism pathways (Fig. S3; Data set S3). MoRpd3 may regulate metabolism pathways, some of which may overlap MoHst4, as well as phosphatidylinositol signaling systems, RNA transport, aminoacyl-tRNA biosynthesis, ubiquitin mediated proteolysis, and nucleotide excision repair, based on KEGG enrichment of DEGs between *RPD3OX* mutant and the WT strain (Fig. S3; Data set S3).

10.1128/mSphere.00118-21.2 FIG S2 GO enrichment of DEGs. (A) GO enrichment of DEGs in *mohst4*Δ mutant versus wild type. (B) GO enrichment of DEGs in *RPD3OX* strain versus wild type. GO terms were cataloged as cellular component, molecular function, and biological process, represented by different colors. The yellow line represents the threshold of *P = *0.05. The GO term list of the top 20 is denoted in the panel on the right. Download FIG S2, TIF file, 2.2 MB. Copyright © 2021 Lin et al. 2021 Lin et al. https://creativecommons.org/licenses/by/4.0/ This content is distributed under the terms of the Creative Commons Attribution 4.0 International license.

10.1128/mSphere.00118-21.3 FIG S3 KEGG enrichment of DEGs. (A) KEGG enrichment of DEGs in *mohst4*Δ mutant versus wild type. (B) KEGG enrichment of DEGs in *RPD3OX* strain versus wild type. The yellow line represents the threshold of *P = *0.05. The pathway list of the top 20 is denoted in the panel on the right. Different colors represent different A class. Download FIG S3, TIF file, 2 MB. Copyright © 2021 Lin et al. 2021 Lin et al. https://creativecommons.org/licenses/by/4.0/ This content is distributed under the terms of the Creative Commons Attribution 4.0 International license.

10.1128/mSphere.00118-21.7 DATA SET S2 GO enrichment of differentially expressed genes (DEGs). (Sheet 1) The GO enrichment of DEGs between the *mohst4*Δ (KO) mutant and the WT strain. (Sheet 2) The GO enrichment of DEGs between the *RPD3OX* (OX) strain and the WT strain. Download Data Set S2, XLSX file, 0.03 MB. Copyright © 2021 Lin et al. 2021 Lin et al. https://creativecommons.org/licenses/by/4.0/ This content is distributed under the terms of the Creative Commons Attribution 4.0 International license.

10.1128/mSphere.00118-21.8 DATA SET S3 Pathway enrichment of differentially expressed genes (DEGs). (Sheet 1) KEGG enrichment of DEGs between the *mohst4*Δ (KO) and WT strains. (Sheet 2) KEGG enrichment of DEGs between the *RPD3OX* (OX) strain and the WT strain. Download Data Set S3, XLSX file, 0.03 MB. Copyright © 2021 Lin et al. 2021 Lin et al. https://creativecommons.org/licenses/by/4.0/ This content is distributed under the terms of the Creative Commons Attribution 4.0 International license.

Next, we searched for conidiation-related genes among the DEGs (*mohst4*Δ versus WT, *RPD3OX* versus WT, respectively) to gain insight into the potential mechanism(s) involved in such HDACs’ regulation of conidiation. In the filtered DEGs (FDR < 0.05 and |log_2_FC| > 1; Data set S1), only a negative regulator of conidiation, encoded by *CNF3* (30) (ID ncbi_5050289 in Data set S1), was found to be significantly downregulated in the *RPD3OX* strain. This suggests that the hyperconidiation phenotype in the *RPD3OX* strain may be caused by transcriptional suppression of *CNF3*. To screen for more conidiation-related genes potentially under MoHst4 and/or MoRpd3 regulation, we searched in the unfiltered annotated DEGs (data not shown). DEGs with the expression rates (mutant/WT) falling into the range of |log_2_FC| > 0.5 were also taken into consideration. Several Zn_2_Cys_6_ transcription factors encoding genes (30) were differentially regulated in the *mohst4*Δ mutant (Table 1). Two reported conidiation-related genes, *CON7* (31) and *CON8* (32), were downregulated, whereas another conidiation-specific gene, *VELC* (33), was upregulated in the *mohst4*Δ mutant. We infer that altered expression of these functional genes in the *mohst4*Δ mutant may be a possible reason for the observed reduction in conidiation therein. In the *RPD3OX* mutant, two transcription factors, Cnf3 and Cnf4, both as negative regulators of conidiation (30), were downregulated (Table 1). Two conidiation-related genes, *CON6* and *CON8*, were upregulated in the *RPD3OX* strain (Table 1), which may also contribute to increased conidiation in this mutant. We also noticed that two metabolism genes, *CreC* and *Nit-4*, were downregulated in the *RPD3OX* mutant (Table 1). Both *CreC* and *Nit-4* were reported to positively regulate *M. oryzae* conidiation (30, 34), but their downregulation did not cause reduced conidiation in the *RPD3OX* mutant, possibly because the function of transcription factors Cnf3 and/or Cnf4 was more predominant. *CreC* was also required for full virulence of *M. oryzae* (34); therefore, its downregulation in the *RPD3OX* strain may also (at least partially) contribute to the loss of pathogenicity.

**TABLE 1:** Conidiation-related genes potentially regulated by MoHst4 or MoRpd3

Gene ID	Conidiation in null mutant	Description	log_2_(fc)	FDR
KO/WT				
*MGG_05287* (ncbi_2675319)	Increased	Transcription factor Con7	−0.645285588	0.00169436
*MGG_00513* (ncbi_2674755)	NA^*a*^	Con8	−0.870989433	0.545154889
*MGG_13350* (ncbi_5050282)	Reduced	Transcription factor Fzc57	−0.719655709046125	0.129827360337284
*MGG_14719* (ncbi_5049346)	Reduced	Velc (velvet protein)	1.185386171	0.072307818
*MGG_14852* (ncbi_5048888)	Reduced	Transcription factor Fzc61	0.634471171636212	0.264486970308082
*MGG_06626* (ncbi_2684781)	Reduced	Transcription factor Fzc37	1.9218121077834	9.3186125844172E−10
*MGG_08185* (ncbi_2678301)	Reduced	Transcription factor Fzc45	1.05185228892067	0.199813318625697
*MGG_00494* (ncbi_2674824)	Reduced	Transcription factor MoPro1	1.1543697704616	0.315362823373482
*MGG_05659* (ncbi_2676015)	Reduced	Transcription factor CCA1	0.746243407754218	0.428697770251771
*MGG_01569* (ncbi_2679515)	Increased	Transcription factor MoYcp4	0.923535813120965	0.203020066183811
OX/WT				
*MGG_13360* (ncbi_5050289)	Increased	Transcription factor Cnf3	−3.359895945	0.021517936
*MGG_01043* (ncbi_2674379)	Reduced	Catabolite repression protein creC	−1.057536888	0.16163822
*MGG_03183* (ncbi_2676556)	Increased	Transcription factor Cnf4	−0.903162329	0.285379653
*MGG_01518* (ncbi_2679161)	Reduced	Nitrogen assimilation transcription factor nit-4	−1.219948418	0.273679551
*MGG_02246* (ncbi_2681343)	Increased	Con6	0.540568381	0.265452853
*MGG_00513* (ncbi_2674755)	NA^*a*^	Con8	1.000549982	0.274300794

a NA, not assessed.

The *RPD3OX* was defective in conidial death (Fig. 4 and 5), which is critical for appressorium-mediated host invasion and disease symptoms (27). Therefore, we next searched for the genes related to cell death among the DEGs in *RPD3OX* versus WT as a possible mechanism underlying MoRpd3 regulation of *M. oryzae* pathogenicity. We noticed that 17 genes related to lipid peroxidation and 3 genes related to iron homeostasis, two important aspects for inducing ferroptosis, were differentially regulated in the *RPD3OX* strain (Table 2). This result suggests that MoRpd3 likely regulates *M. oryzae* pathogenicity by modulating the ferroptotic cell death pathway in conidia.

**TABLE 2:** MoRpd3 downstream genes potentially related to ferroptotic cell death

Gene ID	Description
Lipid peroxidation	
*MGG_07210* (ncbi_2683098)	Cytochrome P450 6A1 (Diaporthe helianthi)
*MGG_03933* (ncbi_2677163)	Phosphatidylinositol *N*-acetylglucosaminyltransferase GPI3 subunit (Magnaporthe oryzae 70-15)
*MGG_14057* (ncbi_5051416)	Lipase 5 (Magnaporthe oryzae 70-15)
*MGG_17385* (ncbi_12984055)	Dolichyl pyrophosphate phosphatase (Magnaporthe oryzae Y34)
*MGG_14244* (ncbi_5048803)	Lipase (Colletotrichum graminicola M1.001)
*MGG_08919* (ncbi_2679905)	UDP-glucose, sterol transferase (Magnaporthe oryzae 70-15)
*MGG_00220* (ncbi_2674858)	NADP-dependent alcohol dehydrogenase 6 (Magnaporthe oryzae 70-15)
*MGG_04014* (ncbi_2677447)	Dihydroxyacetone kinase (Magnaporthe oryzae 70-15)
*MGG_06174* (ncbi_2684316)	Phospholipase D active site-containing protein (Magnaporthe oryzae 70-15)
*MGG_09481* (ncbi_2680552)	Leukotriene A-4 hydrolase (Magnaporthe oryzae 70-15)
*MGG_06143* (ncbi_2684255)	Lysophospholipase NTE1 (Magnaporthe oryzae 70-15)
*MGG_06660* (ncbi_2684833)	3-Oxoacyl-(acyl-carrier-protein) reductase (Magnaporthe oryzae 70-15)
*MGG_10879* (ncbi_2676401)	Bifunctional P-450:NADPH-P450 reductase (Magnaporthe oryzae 70-15)
*MGG_10005* (ncbi_2681028)	Glycerol kinase (Magnaporthe oryzae Y34)
*MGG_05349* (ncbi_2675838)	Acyltransferase (Scedosporium apiospermum)
*MGG_02921* (ncbi_2682474)	Aldose reductase (Magnaporthe grisea)
*MGG_01824* (ncbi_2679201)	Proapoptotic serine protease NMA111 (Magnaporthe oryzae 70-15)
Iron homeostasis	
*MGG_00133* (ncbi_2674429)	Siderophore iron transporter *mirC* (Magnaporthe oryzae 70-15)
*MGG_02158* (ncbi_2681069)	Plasma membrane iron permease (Magnaporthe oryzae 70-15)
*MGG_07220* (ncbi_2683108)	Iron transport multicopper oxidase FET3 (Magnaporthe oryzae Y34)

Overall, by utilizing comparative transcriptomics, we identified potential downstream genes epigenetically regulated by *MoRPD3* and/or *MoHST4*. Our results suggest that metabolism and signaling pathways under HDAC I and/or III complex regulation are important for *M. oryzae* conidiation and pathogenesis.

### *MoHST4* and *MoRPD3* function in response to stress conditions.

**FIG 6:**
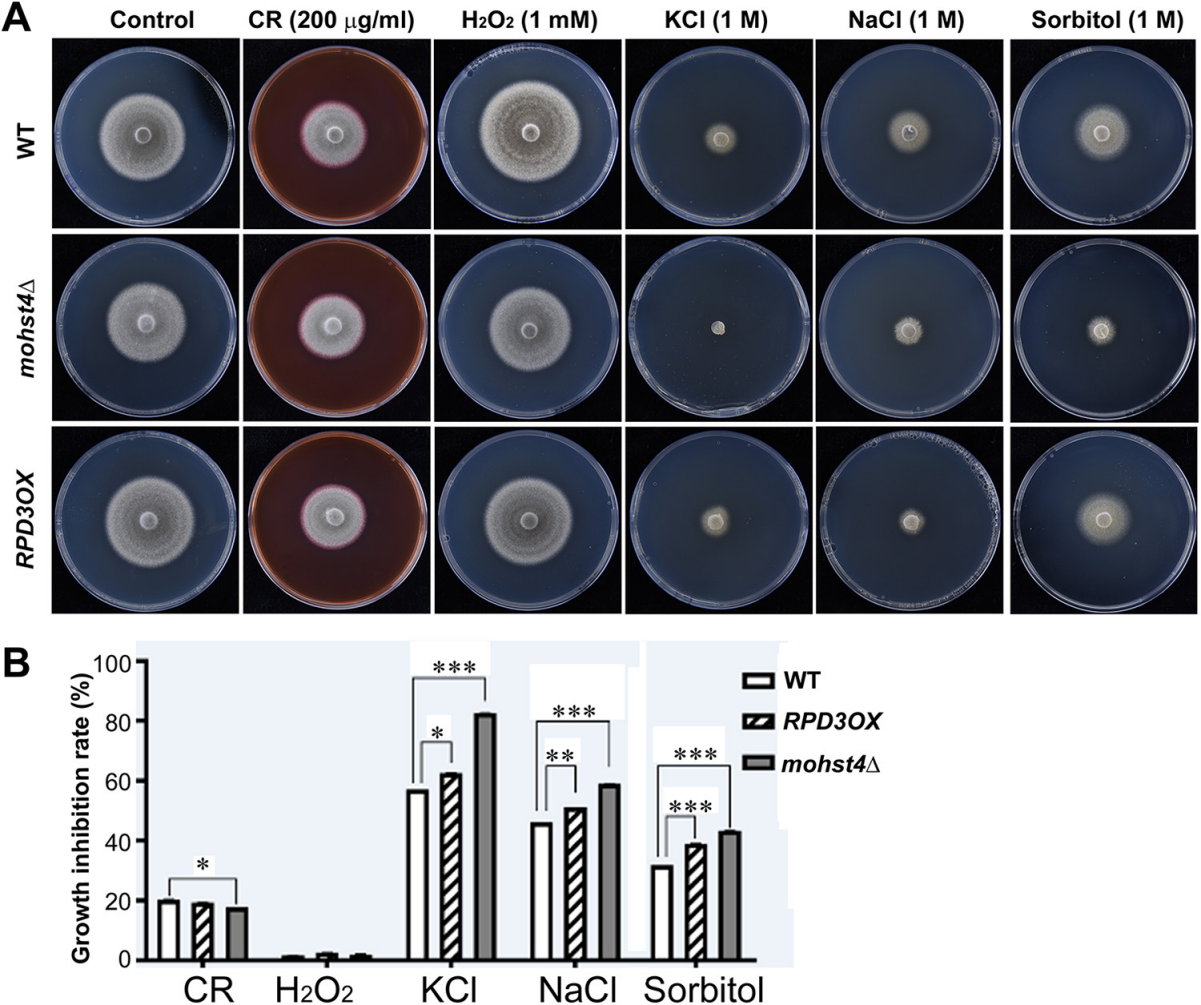
Stress tolerance assays. (A) Colony morphology of WT, the *mohst4*Δ mutant, or the *RPD3OX* strain under diverse stress conditions. The mycelial cultures were grown at 28°C (dark) for 6 days before photographs were taken. (B) Growth inhibition rates were calculated based on reduction of colonial diameter. Means ± SE were derived from three biological repeats of the experiment, each containing 3 colonies. *, *P < *0.05 versus WT; **, *P < *0.01 versus WT; ***, *P < *0.001 versus WT.

We further evaluated the stress tolerance in the *RPD3OX* and *mohst4*Δ mutants in comparison to that in the wild-type strain by assessing their mycelial growth on complete medium (CM) supplemented with salt stress (1 M NaCl or 1 M KCl), the osmotic stress (1 M sorbitol), oxidative stress (1 mM H_2_O_2_), or cell wall-perturbing reagents (200 μg/ml Congo red [CR]) (Fig. 6A). Quantification of the growth inhibition rate based on colony diameter showed that *RPD3OX* and *mohst4*Δ mutants displayed a growth inhibition rate (%) significantly higher than that of the wild type under a range of stress conditions (Fig. 6B). This result showed that MoRpd3- and MoHst4-catalyzed histone remodeling also plays a role in stress tolerance in *M. oryzae*.

## DISCUSSION

Histone acetylation/deacetylation mediated by histone acetyltransferases/deacetylases has been well studied in filamentous fungi. The histone acetyltransferase Gcn5 belongs to the GNAT (Gcn5-related *N*-acetyltransferases) family. In Cryptococcus neoformans, Gcn5 regulates the expression of multiple glucosidases, including Kre61, which is involved in cell wall biosynthesis and capsule architecture (35), thereby allowing C. neoformans to respond appropriately to the host during infection process (36). In *M. oryzae*, light induces autophagy by triggering the cleavage and nuclear translocation of the Gcn5 protein, thus removing Atg7 acetylation to promote conidiation (37). In recent years, HDACs of filamentous fungi were found to be decisive regulators of fungal pathogenicity and production of important fungal metabolites such as antibiotics and toxins (38). In Aspergillus fumigatus, the HDAC1 inhibitor can effectively suppress spore production and germination, thus inhibiting its pathogenicity (6). A recent study characterized a functional Rpd3 HDAC complex in Fusarium graminearum, which plays a function opposite to that of the NuA4 complex containing the HAT FgEsa1 and its interacting protein Fng1 in regulating H4 acetylation and fungal growth (39). In particular, a number of histone deacetylases have been characterized in *M. oryzae* which play various regulatory roles in vegetative growth, conidiation, and host infection (14–18).

In this study, we identified and functionally characterized the orthologous *RPD3* and *HST4* genes in *M. oryzae*. *MoHST4* and *MoRPD3* positively regulated *M. oryzae* conidiation. Although we could not obtain the *rpd3*Δ mutant, we successfully deleted the *SIN3* gene (*MGG_13498*) (Fig. S4A), which encodes the regulatory component of Rpd3 HDAC I (40). The *sin3*Δ mutant was severely defective in mycelial growth (Fig. S4B) and barely survived. Furthermore, the *sin3*Δ failed to produce any conidia but instead showed heavily melanized aberrant structures at the tips of mycelia and aerial hyphae (Fig. S4C). These results further support our findings that the Rpd3-Sin3 HDAC I is, indeed, a strong positive regulator of *M. oryzae* conidiation. The native *in locus* tagged Sin3-GFP signal was predominantly in the nucleus during all stages of asexual and invasive development in *M. oryzae* (Fig. S4D). We infer that the MoRpd3 HDAC function occurs in the nucleus together with the Sin3 component. The cytosolic MoRpd3 likely modifies nonhistone protein targets.

10.1128/mSphere.00118-21.4 FIG S4 Functional characterization and subcellular localization of the Rpd3 HDAC component Sin3 in *M. oryzae*. (A) Southern blotting to verify the deletion of *SIN3* gene. Genomic DNA of the indicated strains were digested with *Kpn*I and probed with the PCR-amplified 5′-UTR fragment (primers listed in Table S1). The presence of the 3.5-kb band in the WT and its absence in the *sin3*Δ mutant, together with the occurrence of the 4.5-kb band, confirmed the successful replacement of *SIN3* locus with *HPH^+^*. (B) Wild-type *M. oryzae* or the *sin3*Δ mutant was cultured on prune agar medium at 28°C prior to being photographed at 5 dpi. Scale bar represents 1 cm. (C) Photomicrographs depicting melanized structures at the tips of mycelia or aerial hyphae of the *sin3*Δ mutant, which failed to produce conidia. Scale bar represents 10 μm. (D) Confocal microscopy-based visualization of Sin3-GFP, which was predominantly nuclear in aerial hypha/conidiophore, conidia, and the penetration/invasive hyphae in *M. oryzae*. Scale bar represents 5 μm. Download FIG S4, TIF file, 2.5 MB. Copyright © 2021 Lin et al. 2021 Lin et al. https://creativecommons.org/licenses/by/4.0/ This content is distributed under the terms of the Creative Commons Attribution 4.0 International license.

Cell biology-based analysis showed that conidiophore formation seemed normal in the *mohst4*Δ and the *RPD3OX* strains, with only the conidial numbers borne on a single conidiophore being different from that of the wild-type strain. Our comparative transcriptomics data showed that several conidiation-related genes (30–34, 41) were differentially regulated in the *mohst4*Δ or *RPD3OX* strain (Table 1). Most of these genes were shown to be involved in conidia formation but not in conidiophore morphology, consistent with the phenotype observed in these two aforementioned strains. Unexpectedly, positive regulators of conidiation, except for Fzc57, were upregulated, despite conidiation being highly reduced in the *mohst4*Δ mutant. We infer that either Fzc57 is a predominant regulator of conidiation or other unidentified Hst4-specific conidiation-related gene(s) determine the phenotype observed in the *mohst4*Δ mutant.

Using comparative transcriptomics, we also identified the potential downstream pathways under MoRpd3 regulation, namely, RNA transport, biosynthesis of amino acids (phenylalanine, tyrosine, and tryptophan), and carbohydrate metabolism (fructose and mannose). GO enrichment showed that HDAC I (containing MoRpd3) and HDAC III (containing MoHst4) differentially regulated downstream genes/pathways, as targets of MoRpd3 are mainly cytoplasmic components, while those of MoHst4 are associated with membrane compartments. We were interested in screening for the common downstream regulatory genes/pathways potentially related to fungal sporulation/conidiation and identified seven such common target genes (Data set S1). Among these, the C2H2 zinc-finger transcription factor MGG_07013 was of particular interest. Orthologs of MGG_07013 have been reported in Botrytis cinerea (42) and A. fumigatus (43), wherein both these transcription factors (TFs) play important roles in regulating transcription during fungal sporulation and/or pathogenesis. Future investigations will focus on such candidate genes to fully elucidate and understand the HDAC-driven epigenetic regulation and mechanisms during *M. oryzae* pathogenesis.

As Rpd3 overproduction compromised conidial cell death during appressorium formation/maturation, we were particularly interested in downstream targets of MoRpd3 related to ferroptosis, which was recently reported as being essential for successful infection by *M. oryzae* (28). Among the DEGs in *RPD3OX* versus WT, we noticed 17 genes potentially related to lipid peroxidation (Table 2) that directly contribute to ferroptosis. Moreover, 3 genes were potentially related to iron homeostasis (Table 2), another important contributor to ferroptosis (28). Addition of the ferroptosis inducer FeCl_3_ partially restored pathogenicity in the *RPD3OX* strain. This indicates that MoRpd3 likely regulates conidial ferroptosis to promote appressorium-mediated host infection. On the other hand, we also noticed that DEGs in the *mohst4*Δ mutant were enriched on transmembrane transport, including a variety of metal ions. We infer that the reduced pathogenicity in the *mohst4*Δ mutant may be due at least partially to dysregulated iron homeostasis leading to reduced conidial ferroptosis and appressorium maturation, but such a hypothesis needs to be verified by actual measurement of iron content within the mutant conidia.

Overall, our study revealed biological function and potential downstream genes of MoRpd3 (in HDAC I) and MoHst4 (HDAC III) in *M. oryzae* growth, asexual development, pathogenesis, and stress tolerance. These findings will certainly broaden our understanding of HDAC functions in pathogenic fungi and thus provide a framework for establishing HDAC component(s) as potential target(s) for development of antifungal drugs for control of rice blast and/or other important fungal diseases.

## MATERIALS AND METHODS

### Fungal strains and culture conditions.

The *M. oryzae* wild-type strain B157 (MAT1-2) and all mutant/OX strains used in this study were cultured on complete medium (CM) or prune agar (PA) medium at 28°C. Mycelia used for DNA, RNA, and protein extraction and protoplast isolation were cultured on CM at 28°C with shaking at 180 rpm for 2 days. Protoplast preparation and transformation were conducted as reported previously (44).

For vegetative growth assay, similar-sized plugs of indicated strains were cultivated on PA medium at 28°C and colony diameter/radius was measured after a week. For conidiation, the strains were cultured on PA medium in constant darkness for 3 days followed by growth under 12 h:12 h dark:light cycle for 5 days. Quantification of conidia formation in each strain was performed by counting the total number of conidia in a 5-μl droplet using a hemocytometer under a light microscope, and then production of conidia was normalized to a given unit growth area (100× conidia/cm^2^).

For assessing fungal tolerance toward various stressful conditions, the fungal mycelial plugs were cultured on CM supplemented with Congo red (CR; Genview, 573-58-0; 200 μg/ml), H_2_O_2_ (1 mM), KCl (1 M), NaCl (1 M), or sorbitol (1 M). The fungal cultures were allowed to grow in the dark at 28°C for 6 days prior to photo documentation. The growth inhibition rate (%) for the respective strain under different stress conditions was calculated using the following formula: growth inhibition rate (%) = [diameter (CM) − diameter (stress)]/diameter (CM). All assessments were performed as at least three independent biological repeats, each of which contained three replicates. The statistical analysis was performed using the *t* test formula in Microsoft Excel (version 18.2005.1191.0).

### Plasmid constructs and fungal transformants.

To construct the *MoHST4* gene replacement vector, a 1.2-kb upstream fragment and a 1.2-kb downstream fragment of the targeted open reading frame (ORF) were respectively amplified from the *M. oryzae* genomic DNA. The two flanking sequences were cloned into the *Agrobacterium* transfer-DNA vector pFGL821 to flank the hygromycin B phosphotransferase cassette (*HPH1*), and the resultant plasmid was transferred into the *M. oryzae* wild-type strain B157 through Agrobacterium tumefaciens-mediated transformation (ATMT), following the established protocol (45). All hygromycin-resistant transformants were first screened by locus-specific PCR and then confirmed by Southern blotting.

For generating the *MoHST4*-eGFP (enhanced green fluorescent protein) fusion construct, the fragment containing the full-length coding region of *MoHST4* (except stop codon), along with its native promoter region (1.5 kb), was PCR amplified and in-frame fused with the eGFP ORF at its C terminus. The *MoHST4*-eGFP fusion construct (on pYF11 vector) was transformed into the *mohst4*Δ mutant through PEG-mediated transformation (44). The transformants were evaluated by Southern blotting and examined for GFP signal to verify the complemented strain. To construct the overexpressed eGFP-*MoRPD3*, the eGFP coding sequence was fused in frame to the N terminus of the *MoRPD3* ORF and under the constitutive promoter of the *RP27* locus, on pFGL932 vector (37). The transformed *M. oryzae* was selected by glufosinate resistance, examined for GFP signals under epifluorescence microscopy, and evaluated by qRT-PCR. The primers used for plasmid construction, transformant verification, and RT-PCR and qRT-PCR analyses are listed in Table S1.

10.1128/mSphere.00118-21.5 TABLE S1 Oligonucleotide primers used in this study. Download Table S1, DOCX file, 0.02 MB. Copyright © 2021 Lin et al. 2021 Lin et al. https://creativecommons.org/licenses/by/4.0/ This content is distributed under the terms of the Creative Commons Attribution 4.0 International license.

### Nucleic acid manipulation.

Genomic DNA of each isolate was extracted from vegetative hyphae using the fungal DNA Midi kit (Omega, D3590-01). The resulting genomic DNA was used for PCR screening or Southern blotting hybridization. Southern blotting was performed with the digoxigenin (DIG) high prime DNA labeling and detection starter kit II (Roche, Mannheim, Germany). Ten μg genomic DNA of indicated strains were digested with HindIII/KpnI-HF. Digested products were resolved in a 1% agarose gel and then transferred onto Hybond-N^+^ membrane (Amersham). The 5′ flank sequence of *MoHST4* was used for PCR amplification of the specific probe, which was labeled with digoxigenin-11-dUTP using digoxigenin (DIG)-high prime, and then hybridization and detection were performed according to the instruction manual (Roche Applied Science).

For verification of *MoRPD3* overexpression, total RNA was isolated from liquid CM-cultured fungal mycelia using the RNeasy minikit (Qiagen, 74104). For monitoring *MoHST4* and *MoRPD3* expression pattern, total RNA was isolated from liquid CM-cultured fungal mycelia, from conidia (freshly harvested), during appressorium formation (12 hpi on inductive surface), and at the invasive growth stage (12 and 24 hpi) using Qiagen RNeasy plant minikit (74104). The TransScript First-Strand cDNA Synthesis SuperMix (TransGen Biotech, catalog number AT301-02) was used for cDNA synthesis. qPCR was performed by using PowerUp SYBR green master mix (Applied Biosystems, A25742), on QuantStudio 6 Flex real-time PCR system (Thermo Fisher Scientific).

For comparative transcriptome analysis, WT, *mohst4*Δ, and *RPD3OX* strains were grown on the PA medium in the dark for 5 days and exposed to light for 12 h before total RNA extraction using the Qiagen RNAeasy minikit (74104). Three biological replicates, each containing growth from 10 petri dishes, were performed for each strain. High-throughput RNA sequencing (RNA-Seq) and transcriptome analysis were performed by Gene Denovo Co. (Guangzhou, China). Short reads were mapped to the complete genome of *M. oryzae* (https://www.ncbi.nlm.nih.gov) using Tophat. Genes with a fold change above 2 and a false-discovery rate (FDR) lower than 0.05 in a comparison of significant differentially expressed genes (DEGs) were subjected to enrichment analysis of Gene Ontology (GO) functions and KEGG pathways, following established protocols (46).

### Total protein extraction and immunoblotting analysis.

For total protein extraction, mycelia grown on CM at 28°C for 1 week were ground with liquid nitrogen to a fine powder and resuspended in 0.5 ml extraction buffer: 10 mM Tris-HCl (pH 7.5), 150 mM NaCl, 0.5 mM EDTA, and 0.5% Nonidet P-40 substitute, with 2 mM phenylmethylsulfonyl fluoride (PMSF) and proteinase inhibitor cocktail (Sigma-Aldrich, cOmplete, 11836170001). Lysates were cleared by centrifugation at 12,000 rpm for 30 min at 4°C. Protein concentration was measured using the Bio-Rad protein assay (500-0006).

For Western blotting, total protein extracts were separated on a 10% SDS-PAGE gel before being transferred to nitrocellulose (NC) membrane. The protein acetylation level was detected using the anti-AcK (1:1,000; Abcam, ab61257) or anti-H3AcK antibody (active motif, 61937). Coomassie blue staining of the total protein served as loading control.

### Pathogenicity assays.

Detached leaves of barley seedlings (cv. Golden Promise) were used for pathogenicity assay. A series of different numbers of conidia of the indicated strains from 10-day-old PA cultures were inoculated onto intact barley leaves and incubated under high humidity (>90%) at 25°C in darkness for the first 24 h, followed by a 16 h:8 h, light:dark cycle. For infection assays using rice (cv. LTH) leaf explants, the wild-type or *RPD3OX* conidia in water or FeCl_3_ solution (5 μM) were inoculated, 0.0004% HCl serving as solvent control. The inoculated leaves were kept under high humidity (>90%) at 25°C in darkness for the first 24 h, followed by a 16 h:8 h, light:dark cycle, for 4 to 5 days before photographing.

The rice seedlings (cv. LTH) used for pathogenicity assays were at three- to four-leaf stage and sprayed with 5 ml conidial suspension (2 × 10^5^ conidia/ml) for each pot containing 5 to 6 seedlings. Each assay was repeated thrice. The blast disease lesions were evaluated and photographed at 5 to 7 dpi.

### Staining and microscopy.

The eGFP-MoRpd3 signal was observed and imaged in *M. oryzae* conidia, using an Axio Observer Z1 microscope (Zeiss, Jena, Germany) equipped with sCMOS camera (PCO Edge, Kelheim, Germany).

Conidial viability was assessed by trypan blue (Sigma; T6146; 1%) staining, following an established protocol (28). Microscopic examinations and imaging were performed using an Olympus BX53F microscope (Olympus, Japan) equipped with a digital single-lens reflex camera (Canon, DS126571, Japan).

### Data availability.

All requisite data from this study are openly available from public repositories and/or online databases as indicated in the manuscript. Additional data may be found in the supplemental material files.
