# Supplementary figures and images for: The Histone Deacetylases MoRpd3 and MoHst4 Regulate Growth, Conidiation, and Pathogenicity in the Rice Blast Fungus Magnaporthe oryzae
Source: mSphere. 2021 Jun 30;6(3):e00118-21. doi: 10.1128/mSphere.00118-21 (PMC8265625; doi:10.1128/mSphere.00118-21)

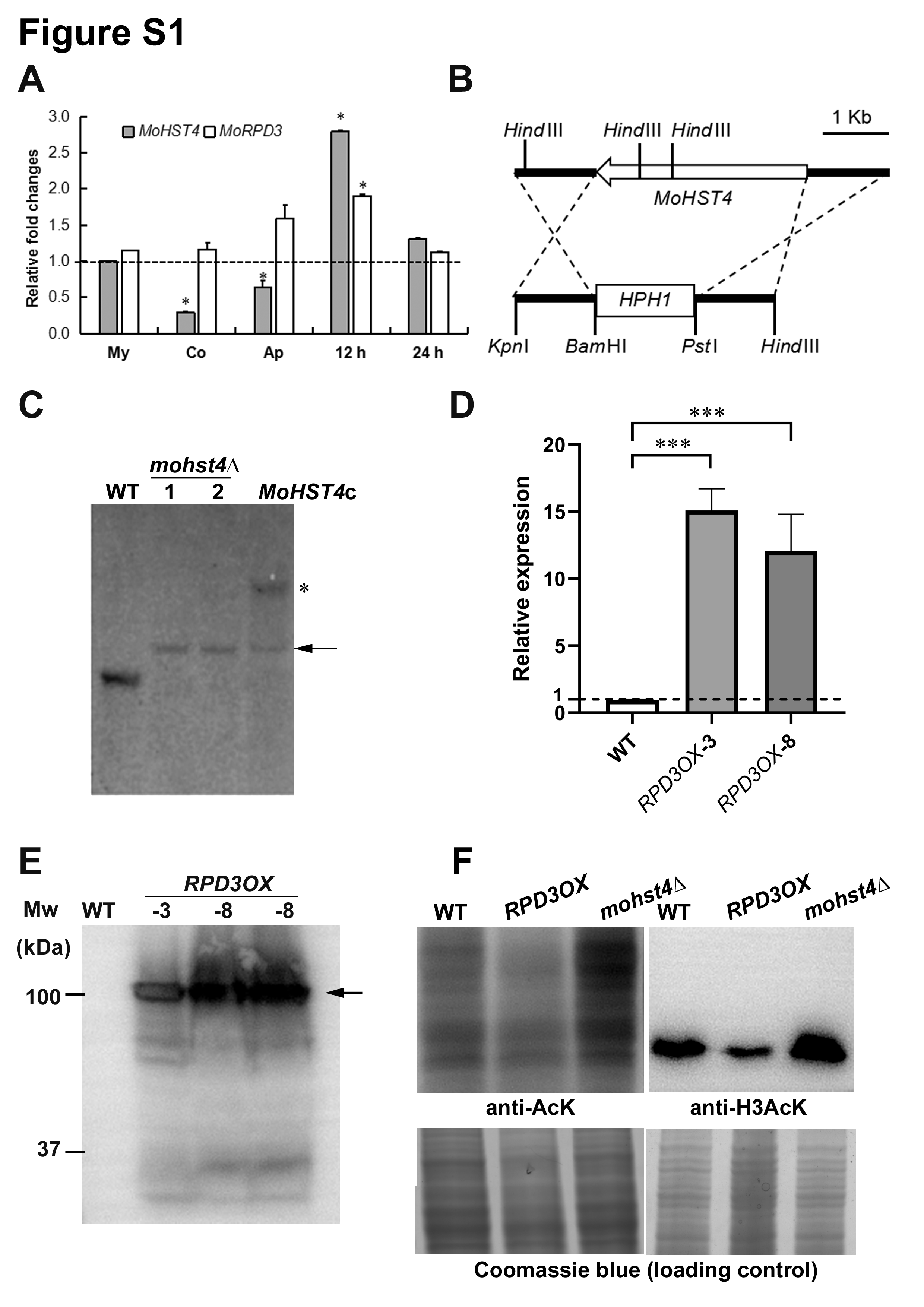

Supplement: FIG S1 [file msphere.00118-21-sf001.tif]

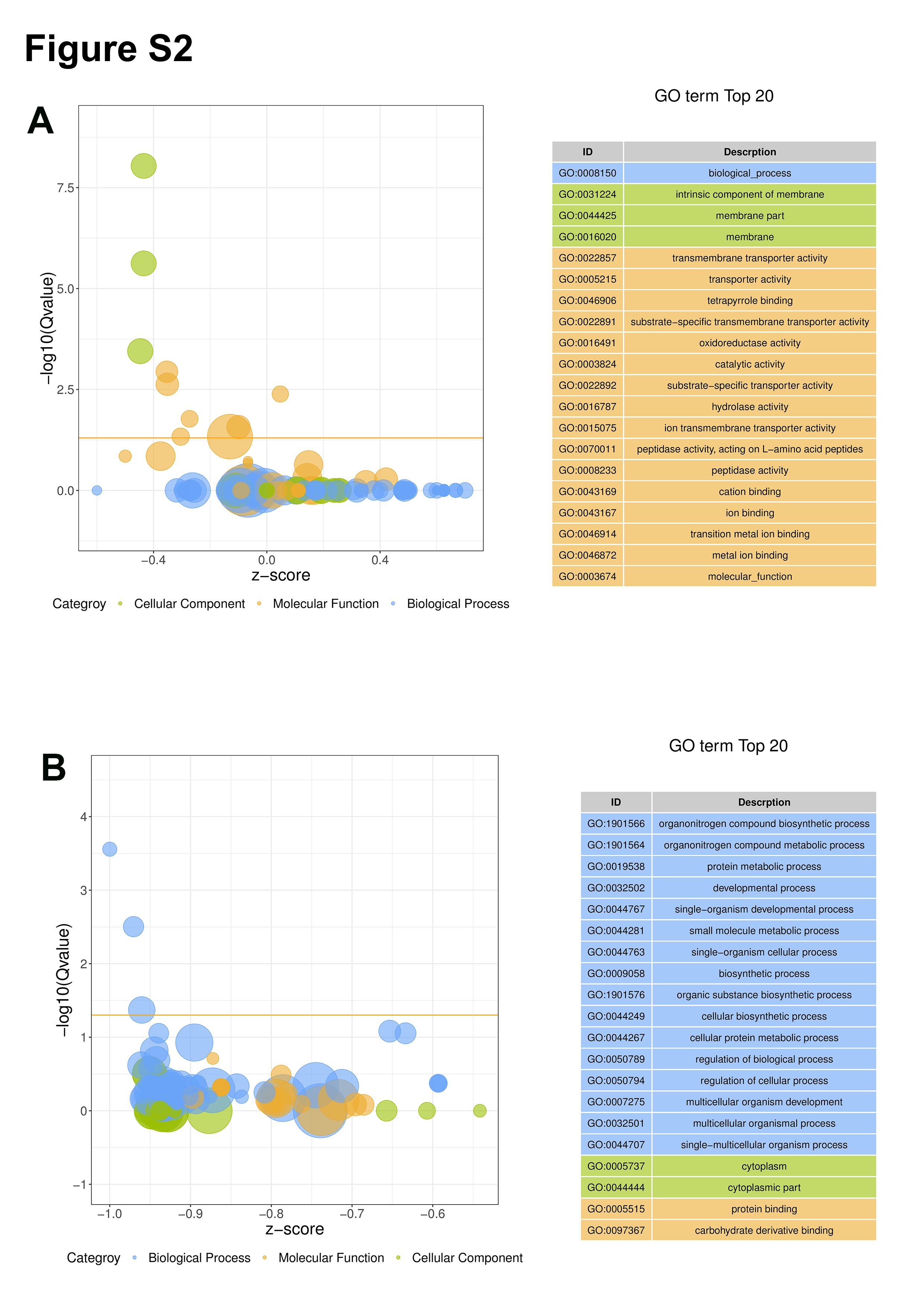

Supplement: FIG S2 [file msphere.00118-21-sf002.tif]

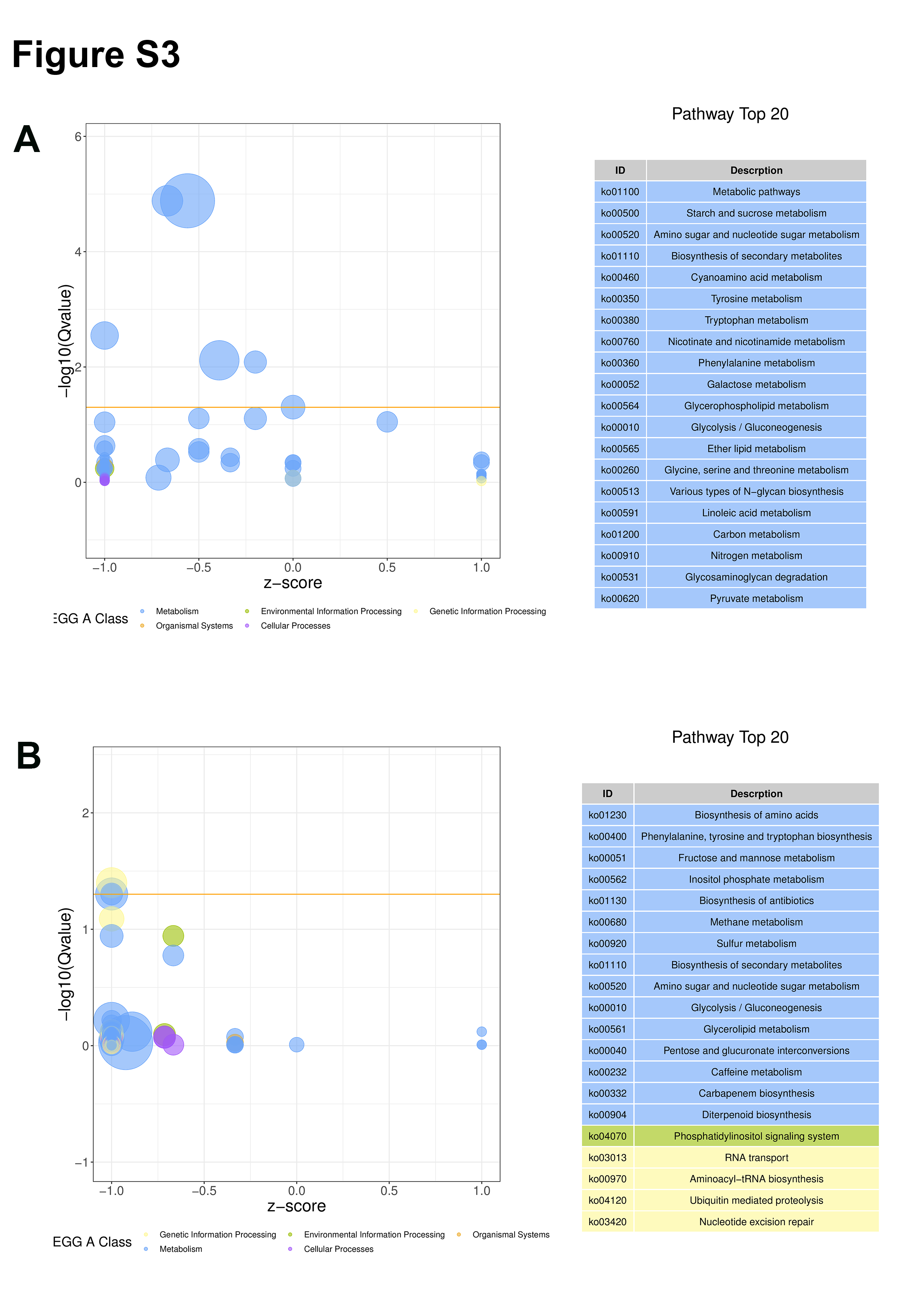

Supplement: FIG S3 [file msphere.00118-21-sf003.tif]

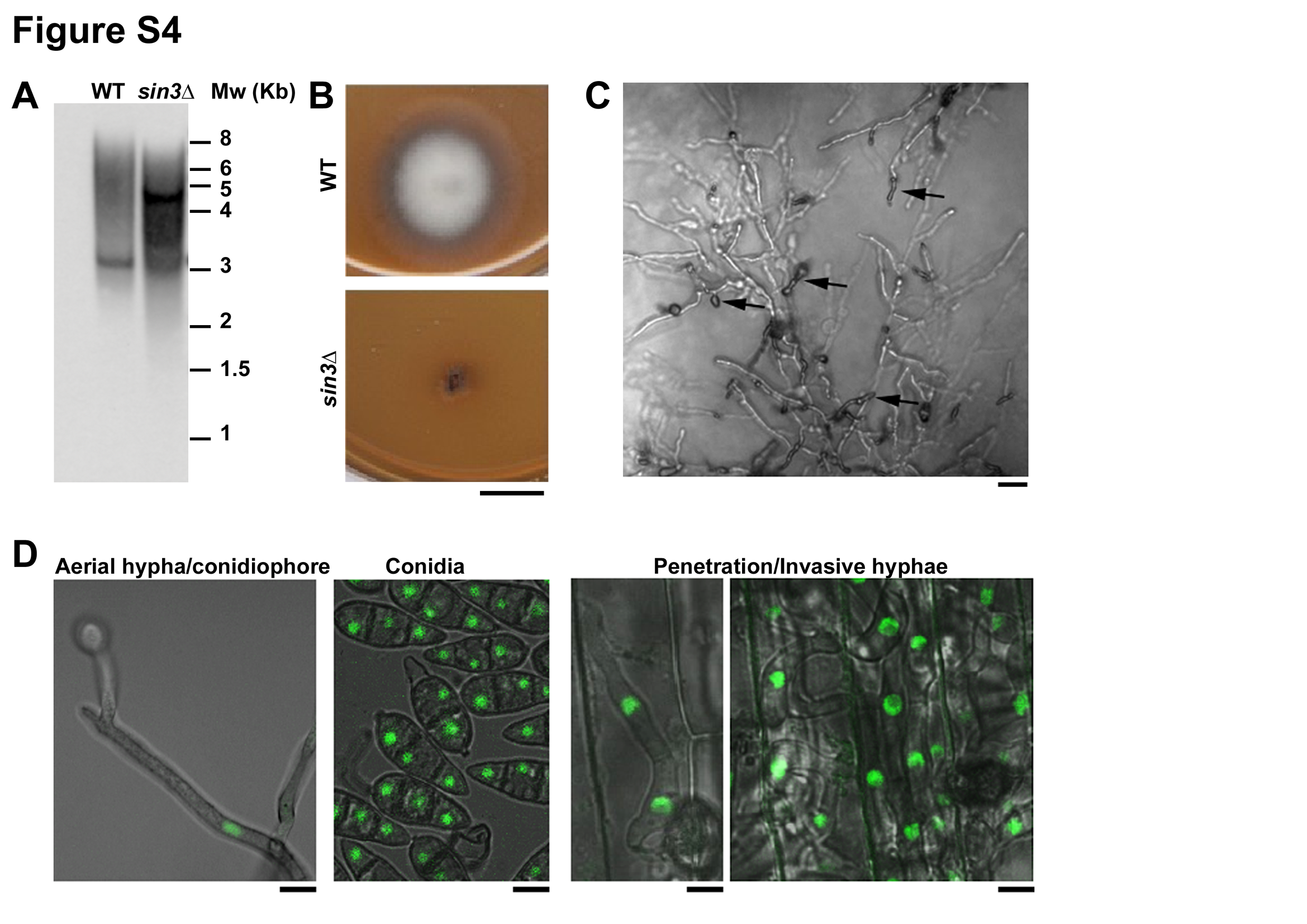

Supplement: FIG S4 [file msphere.00118-21-sf004.tif]
