# Supplementary material for: The Histone Deacetylases MoRpd3 and MoHst4 Regulate Growth, Conidiation, and Pathogenicity in the Rice Blast Fungus Magnaporthe oryzae
Source: mSphere. 2021 Jun 30;6(3):e00118-21. doi: 10.1128/mSphere.00118-21 (PMC8265625; doi:10.1128/mSphere.00118-21)
Supplement: TABLE S1 [file msphere.00118-21-st001.docx]

**Table S1 Oligonucleotide primers used in this study.**

| Gene (Locus) | Description | Enzyme sites | Primer sequence |
| --- | --- | --- | --- |
| *HST4* (*MGG_04588*) | Deletion construct | *Hind*III | 5'-GAGAGTGTT**AAGCTT**CCCTGCCCAGTGCTTGACATTCCA-3' |
|  |  | *Pst*I | 5'-GAGACTGTT**CTGCAG**CGTCAGCTGGCGGGCTGAAGC-3' |
|  |  | *BamH*I | 5'-GAGAGTGA**GGATCC**GCAAACAGTGTTTCCTACGGAGCTGA-3' |
|  |  | *Kpn*I | 5'-AGAGTGA**GGTACC**TTCTGCATTTTGTTTGGCGTATGG |
|  | PCR Verification | — | 5'-AATGCGGTCATGACAGAGTGGG-3' |
|  |  | — | 5'-AGTCGGAGGACGACCTTTTTGC-3' |
|  |  | — | 5'-GTGCTCCTTCAATATCACTCGACCTGC-3' |
|  | Subcellular localization | — | 5'-ACTCACTATAGGGCGAATTGGGTACTCAAATTGGTTCGTTCAGCTCAGAGATTTGACGTGAT-3' |
|  |  | — | 5'-CACCACCCCGGTGAACAGCTCCTCGCCCTTGCTCACGTCGATCAAATGCCCCATAGAC-3' |
|  | Complementation | *BamH*I | 5'-GAGAGTGA**GGATCC**CGTTCAGCTCAGAGATTTGACGTGATTGG-3' |
|  |  | *Kpn*I | 5'-AGAGTGA**GGTACC**TTTGCCAGATAAAGAATATACCACATCAGA-3' |
|  | qRT-PCR | — | 5'-GACCTCGATCCCGAGTTATTTG-3' |
|  |  | — | 5'-GACCATACGCGGACGTAATC-3' |
| *RPD3* (*MGG_05857*) | Overexpression | *Kpn*I | 5'-GACGAGCTGTACAAG**GGTACC**ATGACGGACAAGGCGGGC-3' |
|  |  | *EcoR*I | 5'-CTATGACATGATTAC**GAATTC**TTTCTCATAGTGTATCCAGTCGAACTT-3' |
|  | Verification | — | 5'-CCGACTATACTGAGCACCAACGCGAATTGC-3' |
|  |  | — | 5'-GAATAAACTGTTAGCTTGGATTTTGTTGAA-3' |
|  | qRT-PCR | —  — | 5'-ACAAGGTCGTCAATACGGATAAG -3' |
|  |  |  | 5'-GGATTCGGTGAGGTTTCATAGG-3' |
| *SIN3*  (*MGG_13498*) | Deletion construct | *Kpn*I  *BamH*I  *Hind*III  *Hind*III | \| 5'-GTGT**GGTACC**AGCAGATAAGGCATTTACATG-3' \| \| --- \| \| 5'-GTGT**GGATCC**TGAGCGCCCAGGCGGGCTTTC-3' \| \| 5'-GTGT**AAGCTT**ATTCTTGTCGTTTGCTTTTATC-3' \| \| 5'-GTGT**AAGCTT**ACCGGAGGCACTGTCACAG-3' \| |
|  | Subcellular localization | *EcoR*I  *Kpn*I  *Kpn*I  *BamH*I | 5'-GTGT**GAATTC**AGAGAGGGCAGACAAGCCTG-3'  5'-GTGT**GGTACC**TGCAGAAGACCCAGCACCGC-3'  5'-GTGT**GGTACC**ATGGTGAGCAAGGGCGAGGAG-3'  5'-GTGT**GGATCC**CTACTTGTACAGCTCGTCCATGC-3' |
| *ACTIN* (*MGG_03982*) | qRT-PCR | — | 5'-GTCGCTCTTGACTTTGAGCA-3' |
|  |  | — | 5'-ATACCACCGCTCTCAAGACC-3' |
